# Investigating emerging contaminants in dams from the Vhembe District in Limpopo and, for the first time, in the Orlando Dam, Gauteng Province, South Africa

**DOI:** 10.1007/s11356-026-38342-2

**Published:** 2026-10-27

**Authors:** Mokgadi Flesher Seshoka, Jacobus van Dyk, Irene Jane Barnhoorn

**Affiliations:** 1 Biological Sciences Department, University of Venda, Private Bag X5050, Thohoyandou, 0950 South Africa; 2 Zoology Department, University of Johannesburg, P O Box 524, Auckland Park, ParkJohannesburg, 2006 South Africa

**Keywords:** Antiretroviral drugs, Antibiotics, Herbicides, Insecticides, Fungicides, Metals, Endocrine-disrupting chemicals

## Abstract

Endocrine-disrupting chemicals (EDCs), including certain organophosphate pesticides, herbicides, fungicides, and metals, as well as pharmaceuticals such as antiretroviral drugs (ARVs), are contaminants of increasing concern in freshwater sources in South Africa (SA). One surface water sample from each site was collected from dams in an urban area, Gauteng Province, and from a rural area, the Vhembe District in the Limpopo Province in SA. The water was tested for dolutegravir, nevirapine, tenofovir disoproxil, lamivudine, and efavirenz, human and veterinary pharmaceuticals, insecticides, herbicides, fungicides, and metals. Analytical methods included gas chromatography coupled to time-of-flight mass spectrometry (GC-TOF), liquid chromatography-time-of-flight mass spectrometry (LC-TOF) screening, and liquid chromatography with tandem mass spectrometry (LC/MS/MS). The metals were determined by a semi-quantitative inductively coupled plasma (ICP)-scan. The Orlando Dam screened positive for the five ARVs and two antibiotics, sulfamethoxazole and trimethoprim, and fourteen pesticides were present in the Orlando Dam, two at the Nandoni Dam, one at the TateVondo Dam, three at the Albasini Dam, and two at the Xikundu Weir from the rural area. From 68 metals measured, aluminium (Al), manganese (Mn), and (Zn) were above water quality guidelines. This is the first report of selected ARVs, organochlorine pesticides as EDCs, organophosphate pesticides, herbicides, fungicides, and metals in the Orlando Dam, Gauteng Province, SA.

## Introduction

Human health depends on good water quality and socio-economic development. The rate of human population growth and various anthropogenic activities increase the pollution of available freshwater resources (Mohiuddin et al. 2011; Best 2019; Vardhan et al. 2019). SA has nine provinces, of which the Gauteng Province is the smallest, 18,178 km^2^ (1.5% of the total surface area of SA). Still, it has the highest human population of 16,098,571 (26.3% of SA’s total population). In contrast, the Limpopo Province covers 125,755 km^2^ (10.3%) with a population of 5.9 million people (11% of SA’s total population) (Statistics SA 2023) . The Gauteng Province is primarily urbanized, while the Limpopo Province, which includes the Vhembe District, is mostly rural. Good water quality and socio-economic development are important for human health.

The Vhembe District is one of five district municipalities in the Limpopo Province and has the highest human population of 1,393,949 (Vhembe District Municipality 2022). The Vhembe District lies in the catchment area of the Luvuvhu River, and an array of anthropogenic activities impacts the water quality of this system. Various dams are located within the Luvuvhu River, including the Albasini Dam, Nandoni Dam, the Xikundu Weir, and the TateVondo Dam at the headwaters of the Mutshindudi River. Impacts of anthropogenic activities include the removal of riparian vegetation for subsistence farming, poor agricultural practices, deforestation, and polluted surface runoff. In addition, indoor residual spraying (IRS) using dichlorodiphenyltrichloroethane (DDT) remains an important malaria vector control strategy in the Vhembe District (Barnhoorn 2009). Although DDT has been restricted under Annex B of the Stockholm Convention on Persistent Organic Pollutants (2001) and its production is phased out, an exemption permits its use for IRS for malaria vector control where no suitable alternatives are available (Bouwman 2004). In the river’s upper reaches, closer to the Albasini Dam, plenty of nut trees (macadamia) and fruit (banana, guava, and avocados) orchards contribute to possible insecticide, fungicide, and herbicide pollution. This type of pollution brings about substances that can potentially interfere with humans’ and wildlife’s endocrine systems. The human body uses two systems to coordinate and integrate the functions of the other body systems so that the internal environment remains stable. These systems, the nervous system, and the endocrine system are crucial to health and any disruption to their function can lead to various health effects. The endocrine system is defined by Knight et al. (2021) as “..a series of glands and tissues that produce and secrete hormones, which are used by the body to regulate and coordinate vital bodily functions, including growth and development, metabolism, sexual function, and reproduction, sleep and mood.” EDCs interfere with this process by imitating, inhibiting, obstructing, and even interfering with the normal function of hormones (Hassan et al. 2024). These chemicals can subsequently reach river systems after they are ingested and then excreted by humans. One of the chemicals known to have endocrine-disrupting effects is DDT reported first by Paul Muller in 1938 (Zeng et al. 2024). Although DDT has since been banned by the United States Environmental Protection Agency in 1972, some parts of the world still use it for Malaria control. Before DDT was banned, the Vhembe District Municipality had been implementing Indoor Residual Spraying (IRS) with DDT on mud house walls and roofs to control Malaria transmission since 1945 (Barnhoorn et al. 2009).

The Orlando Dam in Soweto, Gauteng, is located near hospitals and urban housing. Orlando Dam is situated in the Klipspruit wetland catchment area. The Soweto township has a population of 1,895,921 (Statista 2023) and covers an area of 10.01 km^2^. Medical and other wastes impact the Klipspruit and the larger Klip River catchment area and effluent from wastewater treatment plants (WWTPs) (Personal observation, All authors; Bengu et al. 2017). Although no commercial crop/fruit/nut farming is being practiced here, there are informal, small-scale farming practices and herds of goats, sheep, and cattle that graze the catchment area near the Orlando Dam. Solid waste pollution, including personal hygiene products, used contraceptives, and plastics, is especially common in this urban catchment area (personal observation: MF Seshoka and IEJ Barnhoorn).

SA contributes about 22% of the global human immunodeficiency virus (HIV) and acquired immunodeficiency syndrome (AIDS) burden (Statistics SA 2023). Twelve globally approved ARVs have been detected and quantified in South African surface waters: Abacavir, tenofovir, efavirenz, lamivudine, lopinavir, didanosine, stavudine, zidovudine, nevirapine, indinavir, ritonavir, and zalcitabine. These are the most commonly used ARVs in HIV/AIDS treatment therapy and other chronic diseases such as hepatitis C and tuberculosis (Swanepoel et al. 2015; Wood et al. 2015). ARVs can reach river systems via groundwater and surface run-off and this poses a health risk as ARVs have been reported to influence the endocrine system of wildlife and humans; however, there are only a few studies, or research that support this (Gerber, 2019). HIV/AIDS case numbers are higher in urban areas (Gauteng: 1,912,590 people living with HIV; MacDonell and Low 2019) compared to rural areas (Van Schalkwyk et al. 2021), 64 372 people are infected with HIV in Vhembe, which are 19.5% and 1.53% of people infected with HIV in the Limpopo Province and SA, respectively. In the Vhembe District, 5.96% of households have no sanitation systems, while only 15.97% have flush toilets connected to a municipal system. ARVs can, therefore, pollute surface waters because of the limited wastewater treatment.

**Fig. 1:**
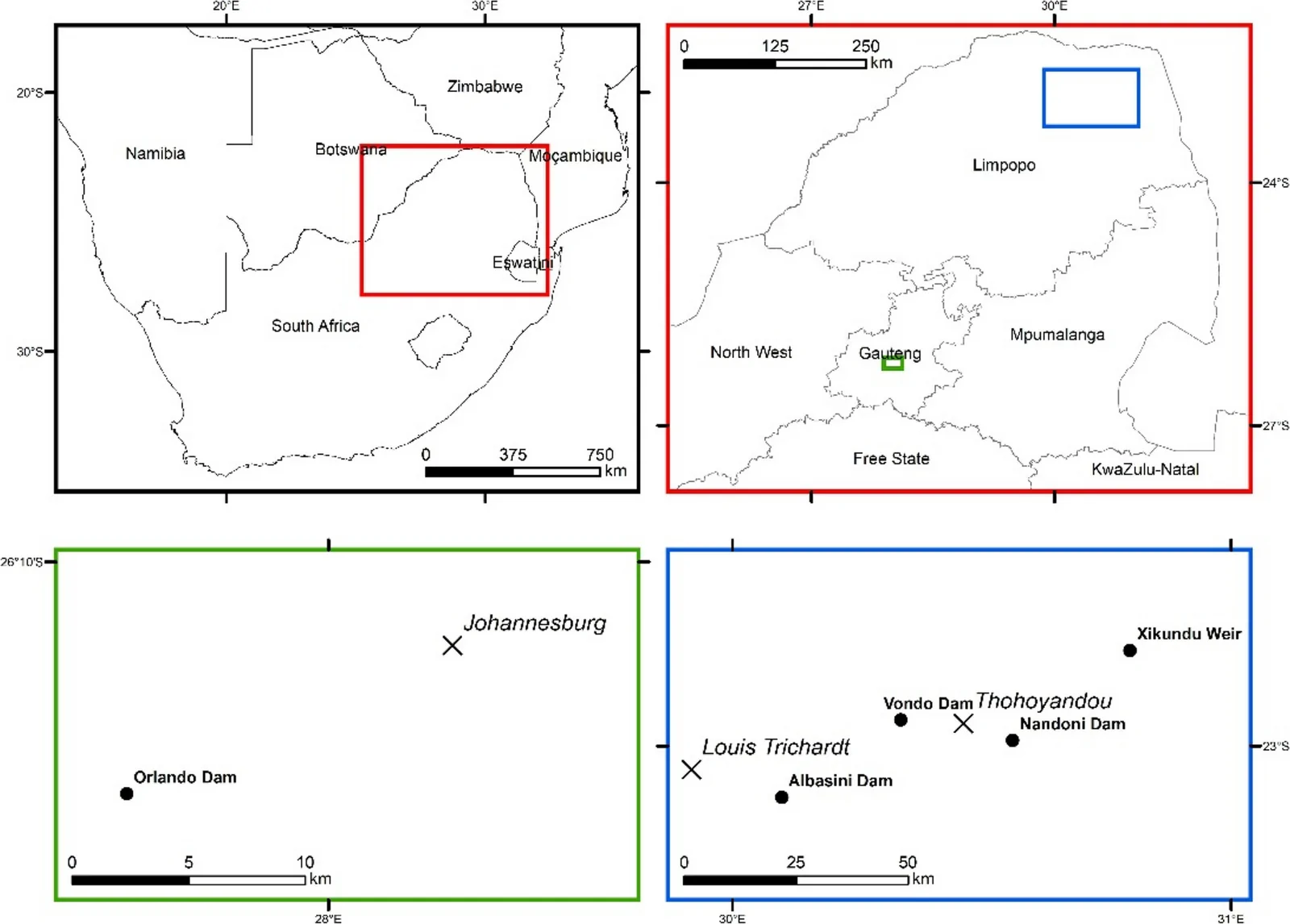
Location of the study area and sampling sites in SA, indicating Orlando Dam, Albasini Dam, Nandoni Dam, TateVondo Dam, and Xikundu Weir

The rural dams in the Vhembe District and the urban dams in Soweto are heavily impacted in the respective provinces. However, limited information is available on the current pollution levels of these surface waters. It is expected that given the differences associated with human activities in rural and urban settings, the water quality will differ and will have different pollution profiles. However, DDT has, for example, previously been reported in urban dams such as Hartbeespoort and Voëlvlei dams in the Northwest and the Cape, respectively (Ansara-Ross et al. 2012), even though this pesticide is only applied in specific rural areas in malaria-infected areas. Metal pollution occurs in both provinces but is hypothesised to originate from different sources. In the Vhembe District, metals are likely derived from agricultural runoff, soil erosion, natural weathering of local geology and domestic wastewater inputs. In contrast, metal contamination in the Orlando Dam is hypothesized to originate primarily from wastewater treatment plant effluent, urban stormwater runoff, historical mining activities within the Witwatersrand Basin and other urban anthropogenic activities. A study by Moyo et al. (2020) reported various metals in water, fish, fruit, and vegetables from the Vhembe District, while Bengu et al. (2017) reported elevated metal concentrations in the water from the Orlando Dam. Exposure to endocrine-disrupting chemicals (EDCs), including selected pesticides and metals, has been associated with adverse health outcomes in both humans and experimental animal models. Reported effects include increased cancer risk (Lion and Olowoyo 2024), metabolic dysfunction associated with environmental contaminant exposure (Hassan et al. 2024), and broader disruption of endocrine function affecting reproductive and developmental health (Javorac et al. 2023). EDCs are of major concern because they have been reported to adversely affect the female reproductive system, contributing to conditions such as early menopause (Grindle et al. 2015), endometriosis (Stephen et al. 2022), and implantation disorders (Tachibana et al. 2007). In addition, exposure to specific organochlorine pesticides (OCPs), such as DDT and its metabolites, has been associated with an increased risk of breast cancer (Rudel et al. 2007). Considering the substantial but different anthropogenic impacts on rural and urban dams, this paper reports on and compares the results of recent water quality analyses for the Orlando Dam in Gauteng Province and the Albasini Dam, Nandoni Dam, TateVondo Dam dams and the Xikundu Weir in the Vhembe District, Limpopo Province, as shown in Fig. 1, with a focus on pesticides (including DDT and its metabolites), pharmaceuticals (including selected ARVs), and metals.

## Materials and methods

### Study site

Water samples were taken from the Orlando Dam in Soweto, Gauteng Province, and from the Albasini, Nandoni, and TateVondo dams, as well as the Xikundu Weir in the Vhembe District of the Limpopo Province, SA, during the summer of 2021. The study sites are shown in Fig. 1.

### Water sampling and preparations

Water samples were collected in 1-L glass bottles for the analysis of organic contaminants and in 500-mL plastic bottles for nutrients and metal analyses. Glass bottles were used to minimize adsorption of organic compounds. In contrast, plastic bottles were used to minimize contamination. Metal samples were preserved with hydrochloric acid (HCl) immediately after collection, before laboratory analyses. The sample container type and volume were selected according to the analytical requirements of the respective analyses. The water was collected approximately 45 cm below the surface. Samples were refrigerated at 4°C until analysis. Results were compared to the target water quality range (TWQR) for domestic use and unpolluted freshwaters as set by the Department of Water Affairs and Forestry (DWAF) (1996a); aquatic ecosystems, DWAF (1996b); drinking water, and the World Health Organization (WHO) (2008).

### Physicochemical parameters and nutrients

The physicochemical water quality parameters, including pH, total dissolved solids (TDS), electrical conductivity (EC), dissolved oxygen (DO), and temperature, were measured in situ using an Eutech multi-parameter meter (Eutech PC 450, Singapore). Nutrients including nitrite, nitrate, total phosphate, orthophosphate, and free and saline ammonia were analyzed by South African National Accreditation System (SANAS) T0391 (ISO/IEC 17025:2005) accredited Waterlab (Pty) Ltd., Pretoria. The method used was a semi-quantitative inductively coupled plasma (ICP)-scan.

### Extraction and screening of organic contaminants

All analyses of organic contaminants were performed by an ISO/IEC 17025-accredited laboratory (FDA Laboratories, Pretoria, South Africa). Water samples were extracted using an in-house solid-phase extraction (SPE) method (GCMS003) prior to instrumental analysis. The extracted samples were screened using gas chromatography coupled to time-of-flight mass spectrometry (GC-TOF) and liquid chromatography coupled to quadrupole time-of-flight mass spectrometry (LC-qTOF). The TOF screening technique provides high sensitivity and accurate molecular mass determination, enabling comparison of exact masses with extensive spectral libraries for the identification of multiple contaminants (Allen and McWhinney 2019; Nurmi and Rantalainen 2012). This approach is suitable for broad-spectrum environmental screening and allows simultaneous identification of numerous organic contaminants.

### GC-TOF analysis of organochlorine pesticides

Selected organochlorine pesticides (OCPs), recognized as endocrine-disrupting chemicals (EDCs), were targeted for analysis. These included α-benzene hexachloride (BHC), β-BHC, γ-BHC, δ-BHC, heptachlor, aldrin, heptachlor epoxide, trans-chlordane, cis-chlordane, α-endosulfan, p,p′-dichlorodiphenyldichloroethylene (DDE), dieldrin, endrin, β-endosulfan, p,p′-dichlorodiphenyldichloroethane (DDD), endosulfan sulfate, p,p′-DDT, and methoxychlor.

Samples were analyzed using an Agilent 7890 gas chromatograph coupled to a LECO TruTOF HT time-of-flight mass spectrometer equipped with a Restek Rxi-5SilMS fused silica capillary column. The oven temperature programme was as follows: initially 45°C, increased to 200°C at 40°C/min and held for 10 min; then increased to 280°C at 20°C/min and held for 5 min; and finally increased to 320°C at 80°C/min and held for 5 min. Helium was used as the carrier gas at a flow rate of 1.0 mL/min. Samples were analyzed using splitless injection in full-scan mode over a mass range of 45–550 m/z. Data acquisition and processing were performed using LECO ChromaTOF GC software (Version 4.41). Compound identification was based on comparisons with the Restek pesticide library and the National Institute of Standards and Technology (NIST) spectral library (Ruiz-Gil et al. 2008).

### LC-MS/MS analysis of pesticides and pharmaceuticals

Targeted pesticide classes included carbamates, fungicides, herbicides, organophosphate pesticides, pesticide synergists, and triazine herbicides. Pharmaceutical screening included quinolones, sulphonamides, tetracyclines, macrolides, thyreostats, β-agonists, β-lactams, anthelmintics, cephalosporins, coccidiostats, tranquilizers, non-steroidal anti-inflammatory drugs (NSAIDs), and other veterinary pharmaceuticals, representing a total of 134 compounds.

Samples were analyzed using an AB SCIEX TripleTOF™ 4600 LC/MS/MS system coupled to an Eksigent MicroLC system fitted with a PAL autosampler. Chromatographic separation was achieved using a Halo C18 column (100 mm × 0.5 mm, 2.7 μm particle size). The mobile phases consisted of distilled water and acetonitrile, each containing 0.05% (v/v) acetic acid. Gradient elution was performed over a 7-min analytical run.

Qualitative compound identification was achieved using quadrupole time-of-flight mass spectrometry (LC-qTOF), whereby exact molecular masses were compared with spectral libraries of known compounds. Compound identity was confirmed by matching the observed parent ions and their characteristic fragment ions generated during tandem mass spectrometry (Ruiz-Gil et al. 2008).

Selected antiretroviral drugs (ARVs), namely dolutegravir, nevirapine, tenofovir disoproxil, lamivudine, and efavirenz, were quantitatively determined using liquid chromatography coupled with tandem mass spectrometry (LC-MS/MS) according to laboratory method LC-MS/MS092.

### ICP analysis of metals

A separate water sample preserved with hydrochloric acid was submitted to a South African National Accreditation System (SANAS)-accredited laboratory (Waterlab, Pretoria, South Africa) for metal analysis. Metal concentrations were determined using a semi-quantitative inductively coupled plasma (ICP) scan to screen for 68 trace elements. The measured concentrations were compared with South African Department of Water Affairs and Forestry (DWAF) water quality guidelines and relevant international guideline values where applicable.

### Quality control

Analyses were performed by ISO/IEC 17025-accredited laboratories using validated analytical methods. Instrument calibration and internal laboratory quality assurance procedures were conducted according to the accredited laboratory protocols. Detection limits for each analytical method are reported with the respective results. Matrix-assisted calibration curves achieved correlation coefficients greater than 0.98, as specified by the analytical laboratory. However, information regarding field blanks, laboratory blanks, duplicate samples, matrix spike recoveries, and certified reference materials was not provided in the analytical reports supplied by the laboratory and is therefore not reported in this study.

## Results

Physical parameters in water are presented in Table 1. The EC and TDS for all the sampling points were within the target water quality range (TWQR) for domestic use and unpolluted freshwaters set by the DWAF et al. (1996a) of 0 to 700 µS/cm. The TWQR for pH for domestic use and aquatic systems is between 6–9 and 6.5–8.5, respectively, whereas the pH guideline of drinking water ranges from 6.5 to 9.5 (DWAF 1996a; DWAF 1996b; WHO 2008). The water pH from Xikundu weir, one site in Nandoni, and two sites in TateVondo dams were marginally above the TWQR for aquatic systems (DWAF 1996b). The pH value from all the dams were within and marginally above the two TWQRs and drinking water guidelines suggested by the WHO in 2008.

**Table 1:** The general water quality parameters measured at three points in the Orlando Dam, Nandoni Dam, TateVondo Dam, Albasini Dam, and the Xikundu Weir

Sampling site	Electrical conductivity (µS/cm)	Total dissolved solids (mg/L)	Temperature (°C)	pH	Dissolved oxygen (mg/L)
Orlando Dam 1	379	243	17.6	7.67	8.4
Orlando Dam 2	377	241	16.9	8.17	8.6
Orlando Dam 3	349	223	16.4	8.17	7.4
Nandoni Dam 1	129.8	83.1	16.4	8.6	*
Nandoni Dam 2	138.5	88.9	17.2	8.2	*
Nandoni Dam 3	142.2	91.1	17.4	8.5	*
TateVondo Dam 1	24.6	15.6	13	8.4	*
TateVondo Dam 2	31.9	20.4	13	8.9	*
TateVondo Dam 3	42.1	26.4	12.7	8.8	*
Albasini Dam 1	184.1	117.4	12.5	8.3	*
Albasini Dam 2	182	116.6	11.6	8.35	*
Xikundu Weir	138.9	88.9	17.5	8.6	*

*Not available

The nutrient levels in the water are presented in Table 2. The nitrate (NO^−^) and nitrite (NO^−^) levels were below the level of detection (LOD) in all dams, with concentrations below 0.2 mg/L. The guideline for drinking water values for total phosphate (PO^3−^), orthophosphate (HPO_4_^2−^), and ammonia (NH_3_) are not established because they occur in drinking water at low concentrations (WHO 2008). The PO ^3−^, HPO_4_^2^⁻, and NH3 were detected at one site in the Orlando Dam (1.2 mg/L, 0.6 mg/L, and 6.5 mg/L respectively). The NH3 levels exceeded the TWQR for domestic use and aquatic ecosystems (DWAF 1996a; DWAF 1996b).

**Table 2:** Selected nutrient values present in the water of the Orlando Dam, Nandoni Dam, TateVondo Dam, Albasini Dam, and the Xikundu Weir

Sampling site	Nitrate mg/L	Nitrite mg/L	Total phosphate mg/L	Ortho-phosphate mg/L	Ammonia mg/L
Orlando Dam	< 0.2	< 0.05	1.2	0.6	6.5
Nandoni Dam	< 0.2	< 0.05	< 0.2	< 0.2	nm
TateVondo Dam	< 0.2	< 0.05	< 0.2	< 0.2	nm
Albasini Dam	< 0.2	< 0.05	< 0.2	< 0.2	nm
Xikundu Weir	< 0.2	< 0.05	< 0.2	< 0.2	nm

Values preceded by < indicate concentrations below the analytical method’s LOD

LOD for nitrate = 0.2 mg/L, nitrite = 0.05 mg/L, total phosphate = 0.2 mg/L, ortho-phosphate = 0.2 mg/L, and ammonia = 0.2 mg/L

*nm* not measured

None of the OCPs, including DDTs, were detected.

The pesticides reported in Table 3 include organophosphate (OP) pesticides, triazine herbicides, herbicides, fungicides, and carbamates. Of the 22 pesticides tested, 19 were below the LOD of 50 ng/L and three were quantifiable. A total of 14 pesticides were detected in the Orlando Dam. Additionally, two at the Nandoni Dam, one at the TateVondo Dam, three at the Albasini Dam, and two at the Xikundu Weir. The atrazine (120 ng/L) found in the water of the Orlando Dam was well below the water standards to protect aquatic ecosystems in SA of 0.01 mg/L (DWAF 1996b) and international standards for pesticides in drinking water for Canada of 0.0018 mg/L (Canadian Council of Ministers of the Environment 2004), as well as Australia 0.0003 mg/L (National Health and Medical Research Council, and Agriculture and Resource Management Council of Australia and New Zealand 1996) and the WHO of 0.00003 mg/L (WHO and IPCS 1996). The Orlando Dam water also contained 86 ng/L metolachlor and 58 ng/L flutriafol. The metolachlor was well below the water standards to protect aquatic ecosystems as described by Canada (0.0078 mg/L) (Canadian Council of Ministers of the Environment 2004), and the international standards for pesticides in drinking water in Australia of 0.3 mg/L (National Health and Medical Research Council, and Agriculture and Resource Management Council of Australia and New Zealand 1996) and the WHO of 0.01 mg/L (WHO and IPCS 1996).

**Table 3:** The pesticides present in the water of the Orlando Dam, Nandoni Dam, TateVondo Dam, Albasini Dam, and the Xikundu Weir

Sampling side	Atrazine (*H*) ng/L	Azoxystrobin (*F*) ng/L	Carbendazim (*F*) ng/L	Diazinon (*I)* ng/L	Diuron (*H*) ng/L	Fluometuron (*H*) ng/L	Flutriafol (*F)* ng/L	Malathion (*I*) ng/L	Metolachlor (*H*) ng/L	Piperonyl butoxide (*PS*) ng/L	Pirimiphos-methyl (*I*) ng/L	Propoxur (*I*) ng/L	Simazine (*TH*) ng/L	Tebuthiuron (*H*) ng/L	Carbaryl (*I*) ng/L
Orlando Dam	120	< 50	< 50	< 50	< 50	< 50	58	< 50	86	< 50	< 50	< 50	< 50	< 50	ND
Nandoni Dam	ND	ND	ND	ND	ND	ND	ND	< 50	ND	ND	< 50	ND	ND	ND	ND
TateVondo	ND	ND	ND	ND	ND	ND	ND	< 50	ND	ND	ND	ND	ND	ND	ND
Albasini Dam	ND	ND	ND	< 50	ND	ND	ND	ND	ND	ND	< 50	ND	ND	ND	< 50
Xikundu Dam	ND	ND	ND	< 50	ND	ND	ND	ND	ND	ND	ND	ND	ND	ND	ND

Values preceded by < indicate concentrations below the analytical method’s LOD (50 ng/L).*ND*, not detected. *H* herbicides, *F* fungicide, *I* insecticide, *PS* pesticide synergist, *TH* triazine herbicide

None of the pharmaceuticals and ARVs tested was detected in the dams from the Limpopo Province. However, the five ARVs and two antibiotics, sulfamethoxazole and trimethoprim, were detected in the water of the Orlando Dam in Gauteng Province (Table 4).

**Table 4:** Pharmaceuticals detected in the water of the Orlando Dam (detection limit = 1 ng/L)

Orlando Dam	Antibiotics	ug/L
	Sulfamethoxazole	1.0
	Trimethoprim	1.0
	**ARVs detected**	**ng/L**
	Efavirenz	1600
	Lamivudine	1700
	Dolutegravir	77
	Tenofovir Disoproxil	< 1
	Nevirapine	< 1

Table 5 presents the trace elements found in each dam. Of the 68 trace elements measured for the following: silver (Ag), arsenic (As), gold (Au), boron (B), beryllium (Be), bismuth (Bi), cadmium (Cd), cerium (Ce), cobalt (Co), chromium (Cr), caesium (Cs), copper (Cu), dysprosium (Dy), erbium (Er), gadolinium (Gd), hafnium (Hf), mercury (Hg), holmium (Ho), indium (In), iridium (Ir), Iron (Fe), lanthanum (La), lithium (Li), lutetium (Lu), molybdenum (Mo), niobium (Nb), neodymium (Nd), nickel (Ni), osmium (Os), lead (Pb), palladium (Pd), praseodymium (Pr), platinum (Pt), rubidium (Rb), rhodium (Rh), ruthenium (Ru), Antimony (Sb), scandium (Sc), selenium (Se), samarium (Sm), tin (Sn), tantalum (Ta), terbium (Tb), tellurium (Te), thorium (Th), thallium (Ti), thulium (Tm), uranium (U), tungsten (W), yttrium (Y), ytterbium (Yb), and zirconium (Zr) were below the detection limit of 0.01 mg/L. Phosphorus (P) was measured in the water of the Orlando Dam, the TateVondo Dam, and the Xikundu Weir at 0.61 mg/L, 0.11 mg/L, and 0.07 mg/L, respectively.

**Table 5:** Metal concentrations in the water of the Orlando Dam, Nandoni Dam, TateVondo Dam, Albasini Dam, and the Xikundu Weir (mg/L)

Metal	Orlando Dam	Albasini Dam	Nandoni Dam	TateVondo Dam	Xikundu Weir
Calcium	24.6	12.04	11	1.50	10.13
Potassium	6.13	2.12	1.50	< 0.5	1.24
Magnesium	9.13	8.79	6.7	1.45	6.36
Sodium	32.4	8.59	9.0	2.18	8.65
Phosphorus	0.61	< 0.01	< 0.01	0.11	0.07
Aluminum	< 0.10	0.42	< 0.10	< 0.10	< 0.10
Barium	0.032	0.05	0.071	< 0.01	0.06
Iron	0.41	< 0.01	0.04	0.04	0.03
Gallium	< 0.01	< 0.01	0.01	< 0.01	0.01
Manganese	0.34	0.04	< 0.03	< 0.03	< 0.03
Silica	0.79	8.83	5.74	2.80	6.22
Strontium	0.06	0.06	0.08	< 0.01	0.07
Titanium	< 0.01	0.02	< 0.01	< 0.01	< 0.01
Zinc	< 0.01	< 0.01	0.01	0.03	0.02

The Al concentration in the water of the Albasini Dam (0.42 mg/L) exceeded the DWAF (1996b) guideline value for the protection of aquatic ecosystems. Barium (Ba) concentrations ranged from 0.03 mg/L in the water from the Orlando Dam to 0.07 mg/L from the Nandoni Dam and were not detected in the TateVondo Dam. The manganese (Mn) levels in the water of the Orlando Dam (0.34 mg/L) and the Albasini Dam (0.04 mg/L) exceed the DWAF guideline for the protection of aquatic ecosystems (0.18 mg/L) (DWAF 1996b). According to Table 5, iron (Fe) was present in the water of the Orlando Dam, Nandoni Dam, TateVondo Dam, and the Xikundu Weir, with a concentration much higher in the Orlando Dam. Gallium (Ga) was found in two dams from the Limpopo Province, the Nandoni Dam and the Xikundu Weir, while silica (Si) was present in all the dams’ water (Table 5). Strontium (Sr) was detected in the four dams monitored, and titanium (Ti) was detected only in the Albasini Dam. The level of zinc (Zn) measured in the Nandoni Dam, TateVondo Dam, and the Xikundu Weir exceeded the DWAF guideline on the protection of aquatic life (0.002 mg/L) (DWAF 1996b).

The overall contaminant profiles observed across the five study sites are summarized in Table 6, highlighting the contrasting contamination patterns between the urban and rural systems.

**Table 6:** Summary of contaminant occurrence across the five study sites

Contaminant class	Orlando (urban)	Albasini	Nandoni	Tate Vondo	Xikundu
Nutrients	●●●	○	○	○	○
ARVs	●●●	–	–	–	–
Antibiotics	●●	–	–	–	–
Herbicides	●●	○	○	○	○
Fungicides	●	–	–	–	–
Organophosphate pesticides	●	●	●	●	●
OCPs (DDTs etc.)	None	None	None	None	None
Metals exceeding guidelines	Mn, Fe	Al	Zn	Zn	Zn

●●● = highest occurrence, ●● = moderate, ● = detected, ○ = below LOD/trace, – = not detected

## Discussion

Overall, the results demonstrate two distinct contamination profiles. The urban Orlando Dam was characterized by a complex mixture of nutrients, pharmaceuticals, antiretroviral drugs, pesticides, and elevated metal concentrations, reflecting wastewater discharges and urban land use. In contrast, the rural dams generally exhibited fewer contaminants, with pesticide detections associated with agricultural activities and isolated metal exceedances. These contrasting profiles indicate that surrounding land use is the primary driver of contaminant occurrence in the study area.

The observations during sampling showed that the Orlando Dam was heavily polluted by fecal matter from livestock, littering with personal hygiene products, sewage, and mining chemical spillage from the upper streams flowing into the dam. Littering with personal hygiene products was unsurprising as the dam is downstream of the Baragwanath Hospital and WWTPs. The pollution status of this dam stood out from all other dams as the results from the study (Tables 1, 2, 3, 4, and 5) indicated higher EC and TDS values, selected pesticides, pharmaceuticals (ARVs and antibiotics), and some metals (Ca, Fe, K, Mn, Na, and P). The dams in the rural setting, the Nandoni, Albasini, TateVondo dams, and the Xikundu Weir, were mainly polluted with garbage and fecal matter from grazing animals. The water was mainly contaminated with organophosphates and carbamates.

The pH level (Table 1) of the Orlando, Nandoni, and Albasini dams and the Xikundu Weir would not affect animal health as they are slightly above the acceptable limit for aquatic health. On the contrary, the pH level of the TateVondo Dam was of concern. The pH of 8.8 and 8.9 of this dam could have been influenced by the garbage deposited by humans observed at the sampling points. However, when compared to the WHO guideline value of between 6.5 and 9.2, the water may be acceptable for drinking by the consumers should there be (Galal-Gorchev et al. 1993).

The tributaries Bailyspruit and Diepkloofspruit rivers of the Orlando Dam are known to be impacted by sewage discharge flow before flowing into the dam (Wepener et al. 2015), which could explain the of PO_4_^3−^, NO_3_^−^, and NH_3_ (Table 2). According to the WHO (2008), the guideline values of NO_3_^−^, NO_2_^−^, and PO_4_^3−^ are 50 mg/L and 3 mg/L, and 0.2 mg/L P (P – provisional guideline value with the evidence of hazard effects) (NO2), respectively. Ammonia exceeded the TWQR for domestic use and aquatic ecosystems (DWAF 1996a, b). According to the WHO (2008), a health-based guideline for drinking water has not been established. Still, levels of NH3 in surface waters are usually below 2 mg/L, which the water of the Orlando Dam exceeded. The level of NH3 was not surprising, as a lot of animal waste and farm animals were observed in the nearby surroundings. However, detectable levels of ammonia, particularly those exceeding the recommended guidelines, are concerning as they have been shown to affect thyroid hormone (elevation of T3, and depletion of T4) and disrupt brain neurotransmission in fish (Gao et al. 2022).

Although the primary focus of this study was on emerging contaminants, selected EDCs, including OCPs, were also investigated, and none of the targeted OCPs was detected at any sampling site. Although found below the LOD, the OP pesticides malathion, diazinon, and pirimiphos-methyl were present in all the dams. At the same time, the carbamates, carbaryl, and propoxur were present in the Albasini Dam and the Orlando Dam, respectively (Table 3). Although OPs act as pest control in agricultural practices, their presence in surface water may be of concern as literature indicated that this group of pesticides may affect the functioning of acetylcholinesterase (AChE) activity, impairing respiration, reproduction, the nervous system, liver, and kidney function in aquatic and terrestrial organisms (Sidhu et al. 2019). Galloway and Handy (2003) and Sebire et al. (2008) reported effects on rodents’ immune systems and fish production, respectively. According to Robb and Baker (2023), OP exposure is a worldwide phenomenon, and about 300,000 deaths occur from an estimated 3 million or more people exposed yearly. Razwiedani and Rautenbach (2017) reported that OP poisoning is a major public health issue in SA. Cases have been reported from the Chris Hani Baragwanath Academic Hospital (Bruins et al. 2019), an intensive care unit in Soweto (Omar et al. 2021), and Cape Town (Davies et al. 2023). It is known that pirimiphos-methyl and malathion are used for malaria vector control in other countries (Haji et al. 2015; Raghavendra et al. 2011), but in SA, malathion is mainly used to control the fruit flies (Diptera: Tephritidae) in citrus orchids and pirimiphos-methyl on stored grains (Manrakhan et al. 2013; Quinn et al. 2011). Botha et al. (2015) reported that carbamate pesticides were responsible for 57 incidents of poisoning of wildlife species between 2009 and 2014, and 19 species were poisoned due to diazinon. In 2022, it was reported that diazinon affected survival, various blood parameters, the gills, and the liver of grass carp (*Ctenopharyngodon idella)* (Vali et al. 2022). The carbamates, carbaryl (insecticide, nematicide, and plant growth regulator), and propoxur (insecticide) present in the water are worrying as these insecticides have a similar mechanism of action at the neuronal synapses and neuromuscular junctions of organisms (Silberman and Taylor 2023). Carbamates have been considered safer because they are readily biodegradable and do not bioaccumulate in the food web (Quinn et al. 2011). However, it has been reported to exhibit high toxicity to organisms in freshwater systems and may affect fish and *Daphnia magna* from 43.19 to 0.031 µmol/L, respectively (Yasser et al. 2015).

From Table 3, atrazine and simazine, two triazine pesticides were detected in the water of the Orlando Dam. Agricultural practitioners widely use it to control grasses in fruit and vegetable orchards (Graymore et al. 2001; Nsibande et al. 2015; Asouzu et al. 2019). Atrazine is also popular for use in SA (Horak et al. 2020) and is among the top 25 used pesticides in SA (Farounbi and Ngqwala 2020), as a control measure against broadleaf and grassy weeds (Nsibande et al. 2015). The 1.20 μg/L atrazine found in Orlando Dam water was below the quality guidelines for protecting aquatic environments in SA of 10 μg/L (London et al. 2005). Although it is said that atrazine poses a “medium risk” (Patterton 2013) when found in drinking water, it must be noted that this herbicide may cause harm to freshwater aquatic species. It has been reported to cause gonadal deformities in frogs (Du Preez et al. 2005a) and reproductive irregularities in zebrafish (Wirbisky et al. 2016). Atrazine is a widely used EDC, and its detection in the present study raises concerns regarding potential endocrine effects in aquatic organisms exposed to chronic concentrations and subsequently humans fishing in these dams (Pathak et al. 2011; Wirbisky and Freeman 2015; Guimarães-Ervilha et al. 2025).

Of the four herbicides found (Table 3), diuron, fluometuron, metolachlor, and tebuthiuron, metolachlor was the only herbicide detected above the LOD at 0.086 mg/L. SA has no official guideline values for metolachlor, but it was higher than the Canadian guideline of 0.0078 mg/L to protect aquatic ecosystems (Canadian Council of Ministers of the Environment 2004). Metolachlor has been reported in rivers from the Western Cape (Curchod et al. 2020), surface waters from the Northwest Province (Du Preez et al. 2005b), and from the Roodeplaat Dam in Gauteng Province of SA (Barnhoorn and van Dyk 2020). Metolachlor is mostly mixed with atrazine to produce effective results on crops. However, Ayansina and Muhammad (2014) reported the death of *Aphanocapsa* (coccoid cyanobacteria) species 2 days after application and the death of inhabiting tadpoles eight days after application. Mathias et al. (2012) reported altered changes in the reproductive endocrinology of male rats and Jin et al. (2011) reported alteration of hypothalamus-pituitary thyroid functioning, both by metolachlor. Of the three fungicides found, flutriafol was detected above the LOD at 58 ng/L (Table 3). However, there is no guideline value for flutriafol in SA. Although herbicides and fungicides have been used since the 1970 s for crop protection, their effects on human and animal health are increasingly being reported (Sparks and Bryant 2021).

Pharmaceuticals such as ARVs have been recognized as hazardous emerging pollutants in the aquatic environment (Swanepoel et al. 2015; Robson et al. 2017). Two types of pharmaceuticals, antibiotics (sulfamethoxazole and trimethoprim) and ARVs (efavirenz, lamivudine, dolutegravir, tenofovir disoproxil, and nevirapine), were detected in the water of the Orlando Dam and reported for the first time (Table 4). According to Ngqwala and Muchesa (2020), persistent antibiotic release may pose risks to humans and animals.

The major concern is efavirenz (1600 ng/L), lamivudine (1700 ng/L), dolutegravir (77 ng/L), tenofovir disoproxil (present), and nevirapine (present) found in the Orlando Dam (Table 4). Studies have indicated that environmentally relevant efavirenz (10.3 ng/L) and nevirapine (3740 ng/L) concentrations may affect nontarget aquatic organisms such as fish (Robson et al. 2017; Nibamureke et al. 2019). Levels of efavirenz have also been detected in South African waters by Gerber (2019) but below the concentrations found in this study. Although the levels of efavirenz were low, they were still cytotoxic to the cells. A low lamivudine concentration of 100 µg/L exposure to *Daphnia magna* (filter feeders) showed a 100% mortality rate at a 48-h exposure, and the *Allium cepa* root tip assay showed an impact on the root lengths (Omotola et al. 2021). These ARVs are of concern as trace concentrations cause human health risks in drinking water (Al-Qaim et al. 2013; Al-Rajab et al. 2018). Although there is increasing concern about contaminants in surface water, there are still limited studies associating these levels with the endocrine effects on humans (Archer et al. 2017). The high concentrations of efavirenz (1600 µg/L), lamivudine (1700 µg/L), and dolutegravir (77 µg/L) together with the antibiotics found are attributed to the high prevalence of HIV/AIDS in SA, especially in the Gauteng Province.

The Al concentration measured in the water from the Albasini Dam was 0.419 mg/L and exceeded the South African Water Quality Guidelines for aquatic ecosystems (DWAF 1996b) (Table 5). Pandey and Jain (2013) suggested that the disruption of the reproduction system in various male models, found in their study was due to Al exposure. Exposure to Al has also been reported to affect reproduction, behavioural, and enzymatic metabolism in planarians (Schaly and Braguini 2024). The form and solubility of Al depend on factors such as pH, alkalinity, and the presence of organic matter. The guidelines indicate that with a pH < 6.5, the Al concentration in water should be ≤ 0.005 mg/L, and with a pH > 6.5, ≤ 0.010 mg/L. The pH of 8.3 of the water of the Albasini Dam was just above alkaline (pH > 8.0) conditions. Although it is presented as soluble in alkaline water, it is not bioavailable to biota (Srinivasan et al. 1999). Although there is no Ti mining in the Vhembe District, 0.017 mg/L was found in the water of the Albasini Dam. Titanium is used as a dye in paint, plastics, enamels, and paper in titanium (IV) oxide, with no guidelines for the safekeeping of aquatic ecosystems. Barium is a naturally occurring metal from weathering rocks and soil with no guidelines for the safe keeping of South African aquatic ecosystems. In the Netherlands, the annual average environmental quality standard (AA-EQS) is 9.3 µg/L and is based on human exposure via fish consumption (Verbruggen et al. 2020). The Ba levels ranged from < 0.01 mg/L in the water from the TateVondo Dam to 0.071 mg/L in the water of the Nandoni Dam and were all above the 9.3 µg/L suggested by Verbruggen et al. (2020). The Fe (0.41 mg/L) and Mn (0.34 mg/L) levels were higher in the water from the Orlando Dam than the other measured water sources. There is no definite TWQR for Fe for the safekeeping of the South African aquatic ecosystems, but the Mn concentration is above the TWQR for aquatic ecosystems of 0.180 mg/L. Although Mn has an essential role in plants and animals, excess amounts may become toxic to animals and affect water quality, therefore disturbing aquatic ecosystems (Wu et al. 2022). Bengu et al. (2017) reported similarly high Fe and Mn concentrations, with Fe ranging from 0.614 to 13 mg/L and Mn ranging from 0.051 to 3.670 mg/L in the water from the Orlando Dam. The higher level of Mn is of concern as it may affect hematology (Ali et al. 2021) and cause genotoxic damage (Tuzuki et al. 2017). The Orlando Dam receives water from the Diepkloofspruit and the Baileyspruit rivers, which receive water runoff from groundwater originating from the Witwatersrand Basin, highly contaminated with toxic metals (Chetty et al. 2021).

Silica was measured in all the water tested. Silica is also known as silicon dioxide (SiO_2_) or mineral quartz, and in SA, silica exposure in humans is mainly due to mining (Utembe et al. 2015). However, the earth’s crust comprises approximately 59% of the chemical element Si. Therefore, the levels of Si added to water may be due to the weathering of minerals such as volcanic glass, clays, or quartz (Dürr et al. 2009). Silica, similar to nitrogen and phosphorus, is required for normal plant growth and reproduction. As summarized in Table 6, the overall contaminants profile differed amongst the study sites, with the urban Orlando Dam showing a greater diversity and occurrence of contamination compared with the rural dams and weir.

### Statistical analysis

No inferential statistical analyses were performed because of once-off sampling from each sampling site. Consequently, there were no biological replicates to estimate variability and/or perform statistical analysis. The nutrients, EDCs, pesticides, pharmaceuticals and metals concentrations were evaluated descriptively based on the presence and absence of each measured concentration at each sampling site. Concentrations detected below the analytical method’s limit of detection were reported as < LOD.

## Conclusion

This study provides one of the first integrated screenings of multiple contaminant classes, including nutrients, endocrine-disrupting chemicals, pesticides, pharmaceuticals, antiretroviral drugs (ARVs), and metals, in urban and rural freshwater impoundments in South Africa. Distinct contamination profiles were observed between the urban Orlando Dam and the rural dams of the Vhembe District, demonstrating that land use and surrounding anthropogenic activities strongly influence contaminant occurrence. The Orlando Dam exhibited the highest contaminant burden, characterized by elevated nutrient concentrations, the exclusive detection of five ARVs and two antibiotics, the greatest diversity of pesticides, and elevated concentrations of several metals. In contrast, contaminants detected in the rural dams were largely associated with agricultural activities, particularly pesticide residues and isolated metal exceedances.

Importantly, although organochlorine pesticides, including DDT and its metabolites, were not detected, several other contaminants of emerging concern were present, highlighting the shift from legacy pollutants towards pharmaceuticals, modern pesticides, and trace metals as important freshwater contaminants. These findings demonstrate the value of broad-spectrum chemical screening for identifying contamination sources and supporting water resource management. Continued monitoring, including seasonal sampling and quantitative risk assessment, is recommended to better understand temporal variation, ecological implications and potential human health risks associated with freshwater resources used for recreation, irrigation and domestic purposes.

## Data Availability

Data is available on request.
